# Turkish Stock Market from Pandemic to Russian Invasion, Evidence from Developed Machine Learning Algorithm

**DOI:** 10.1007/s10614-022-10293-z

**Published:** 2022-07-14

**Authors:** Ahmed R. M. Alsayed

**Affiliations:** Department of Economics, Management and Quantitative Methods, Department of Social and Political Sciences, University of Milan, 20122 Milan, Italy

**Keywords:** Coronavirus pandemic, Russian invasion, Elastic net-EMB regression, C22, E44, G12

## Abstract

In recent time, the two significant events; Coronavirus epidemic and Russian invasion are effecting all over the world in various aspects; healthily, economically, environmentally, and socially, etc. The first event has brought uncertainties to the economic situation in most countries based on the epidemic transmission. In addition to that, on 24th February 2022 the Russian invasion of Ukraine affected negatively almost all stock markets all over the world, but the effects are heterogeneous across countries according to their economic-political relationship or neighbourhood, etc. Due to that, the stock market price in Turkey has been affected dramatically over that period. This empirical study is the first attempts to explore the impact of Coronavirus epidemic and Russian invasion on the stock market index XU100 in Turkey by applying the developed statistical method namely elastic-net regression based on empirical mode decomposition which can precisely tackle the nonstationary and nonlinearity data. Then we performed the robustness check by applying a nonlinear techniques Markov switching regression. The data are collected from the beginning of the epidemic in Turkey from March 11, 2020 until May 31, 2022. The finding reveals that there is significant effect of the Coronavirus spreading on the Turkish stock market index, particularly during the first wave. Then after the Russian Invasion the XU100 index is effected more negatively. As the credit default swap and TL reference interest rate have a negative impact but the foreigner exchange rate has a positive significant impact on the XU100 index, and it varies according to the period of short term and long term. Moreover, the results obtained by using the robustness check shows a robust and consistent finding. In conclusion, understanding the impact of Coronavirus pandemic and Russian invasion on the Turkish stock market can provide important implications for investors, financial sectors, and policymakers.

## Introduction

Recently, over the world have been facing an unexpected health epidemic which made a black swan event worldwide at the end of 2019. In addition to that, the Russian invasion to Ukraine started on 24th February 2022, which is the greatest conflict in Europe since the second world war, and it came at a sensitive time with critical economic situation which caused by the Covid-19 epidemic. As a consequence of the invasion at the global economy and financial markets mainly stretch in three channels: economic sanctions, commodities prices, and supply-chain disruptions. As a result of that, the global stock markets have experienced considerable oscillation, but the effects are heterogeneous across countries according to their economic situation and political relationship or neighbourhood to Ukraine or Russian (Boungou & Yatié, 2022). Nevertheless, the invasion generates uncertainties for market and society, as Ukraine is among the top five countries with the value of goods and services in the year 2021. Furthermore, imports and exports relationships between war and non-war countries are also have been affected, causing investor’s uncertainty and negative effects on some of firm’s production, future profitability, expected cash flows, and share prices which leads towards the variations in stock prices.

Undoubtedly, Turkey is one of those affected countries, the first death has been registered on March 17, 2020 by the Ministry of Health in Turkey (MHT). Turkey’s economic market has been collapsed, and market volatility climbed rapidly during the Covid-19 pandemic, besides other economic problems such as weak currency, higher inflation, and unemployment, etc. Also, Turkish sectors have been affected particularly from the countries in which it has an intense trade. Due to that Turkey’s major stock market index Borsa Istanbul 100 (BIST100) has collapsed during the Covid-19 epidemic. Due to that, we considered the presence of Covid-19 pandemic in the forecasting because it is a breaking point for all financial market, and it will provide a good predication for investors and regulators.

The main contribution of this research study is to apply the current developed statistics method namely elastic net regression based on empirical mode decomposition which can tackle precisely the nonstationary and nonlinearity characteristics of the variables and it can tackle the multicollinearity between the predictors, to examine the behaviour and trend of the Turkish stock market (XU100). The predictors are consisting of infected cases of Covid-19, besides the financial Country-level variables namely; credit default swap, foreigner exchange rate USD/TL, and TL reference interest rate.

This research is organized as follows; Sect. 2 represents the background and relative literature review of the Turkish stock market and Russian invasion. Section 3 explains the dataset and the statistical methods. Section 4 illustrates the results and discussion. Section 5 provides recommendations and a conclusion.

## Background and Literature Review of Covid-19 Outbreak and Russian Invasion

Stock markets have been affected rapidly by the Covid-19 outbreak and Russian invasion, as it increased the uncertainty in financial markets at both global and local levels. Depending on this condition, especially emerging countries have been affected negatively by foreign portfolio investment outflows from stock index, as the main stock exchange indices have been collapsed. For example, at the global level, the Dow Jones Industrial Average index has decreased by 29% from the beginning of February 2020 to early of March 2020. Also, Chicago Board Options Exchange Volatility Index has risen more than 80 points in the middle of March 2020, which is reached the highest peak since the global financial crisis in 2008 (International Monetary Fund, 2020).

Moreover, several previous researches have investigated the impact of war or civil conflict on financial markets such as World War II, Gulf War, and the conflict in Yugoslavia, while their findings support that international markets mostly react negatively. However, recent studies investigated the impacts of country-level factors represented by some firms in EU countries and the higher trade countries dependence with Russia during the invasion. The findings support that the negative impacts of Russia’s invasion are more significant for firms headquartered in EU countries or located in regions close to Russia and areas with a huge influx of refugees.

In a nutshell, there are several variables mostly affect the stock market exchange price, which is categorized by three groups; global-level, country-level, and market level (Zhang et al. (2009), and Yang et al. (2018)).

Global-Level Variables: Morgan Stanley Capital International (MSCI) emerging markets index, oil prices, volatility index (VIX), equity market prices and equity indices.

Country-Level Variables: Turkey’s daily Credit Default Swap (CDS), net funding amount of CBRT, securities amount held by CBRT, Treasury bond interest rates and Turkish Lira reference interest rate (TLREF).

Market Level Variables: Equity market traded value, foreign investors’ share in traded value, foreign investors’ share in retention, retention amount of foreign investors in the equity market, and net buying amount of foreign investors.

Furthermore, the literature on the impact of the Covid-19 pandemic on the economic situation has flourished since the beginning of the epidemic. We provide a brief of some researches which examined the changing of economic situations during the pandemic at the global level that provide general view and useful information of the stock market behaviour. As well we provide a brief of some researches which focused particularly on the effect of Turkish economic and stock market prices during the epidemic. As the infected and death cases of Covid-19 could be influenced by other factors in different seasons (Alsayed, 2021).

Gupta and Shaju, (2021) studied the effect of Covid-19 on global financial markets by applying the novel approach to visualize and compare financial markets across the globe using chaos game representation of iterated function systems. They used various data periods of stock markets, then compared while the difference is quantified through a parameter called the proximity index. They found a significant impacts of the financial market across the globe and volatility to the of covid-19 epidemic. Duarte et al., (2021) analysed the effects of public news and historical prices towards the financial losses on the Brazilian stock market. They compared the traditional Buy and Hold and the moving average strategies to several experiments designed by using several machine learning algorithms. The results indicated a strong relationship between news publications and stock price changes in Brazil, suggesting even short-term arbitrage opportunities. Some research studied the effect of financial crises on several pollutant emissions and they have been approved the existence of the relationship between economic and environmental quality (Alsayed et al., 2020). The results showed that financial crises lead to a fall in CO_2_ emissions, as the crises could increase the consumption-based emissions that consumed goods with an inferior environmental quality (Isa et al., 2015).

In addition to that, a few studies have examined the impact of the Covid-19 pandemic on stock market index in Turkey; Kartal et al., (2021) examined the impacts of foreign portfolio flows and monetary policy responses on Turkish stock market with considering the Covid-19 effects. As well they included the foreign exchange rates, credit default swap spreads, and volatility index as control variables, while the stock market index is considered as the dependent variable. They used a daily dataset between January 2, 2017, and October 20, 2020, by applying the nonlinear autoregressive distributed lag model (NARDL), then they checked the robustness by using Markov switching regression (MSR). The sample period is determined from the beginning of 2017 to avoid the uncertainty of market caused by the military coup which happened in July 2016, as the financial market conditions returned to normal in 2017. The findings reveal that foreign portfolio flows have significantly higher effects on the stock market index than that of monetary policy in the Covid-19 period. Yağli, (2020) examined the impact of Covid-19 on the Turkish stock market volatility and compared that situation before and during the period of Covid-19. They detected the transition changing from low volatility during the pre-Covid-19 to high volatility during the Covid-19 period by performing Markov-switching dynamic regression model. The findings supported the existence of significant deterioration in Turkish stock market volatility during the Covid-19 period. Also, the results showed that the increased recovery of Covid-19 infected cases could lead to lower volatility for most industries. In addition, the exchange rate lowers volatility but the credit default swap increased it. Özkan, (2020) investigates the effect of Covid-19 on the Turkish stock market, the data is collected from Borsa İstanbul over the period of June 2019 to July 2020. He applied generalized autoregressive conditional heteroskedasticity to analyse the volatility of financial assets, also he used the Broock, Dechert, Scheinkman (BDS) test to check the nonlinearity in the daily return series. The finding showed that the increase of the Covid-19 cases causing the volatility jump for all sector indices in Turkey particularly in March 2020. Yang et al., (2018) examined the MSCI index emerging market index on the XU100 index since Turkey. The findings support that the MSCI emerging market index would be an important indicator for Turkey as there is positive relationship between the XU100 index and MSCI index, and Turkey is expected to be negatively affected by oil prices. Moreover, volatility index would make a negative effect on main stock exchange XU100 index because the fear on capital markets also increases, and fund outflows from emerging countries increase when volatility index index increases. Due to that, a negative relationship is expected between the XU100 index and the volatility index (Liew et al., 2018). Erdoğan et al., (2019) include foreign exchange rates, Treasury bill, and short-term interest rate as predictor factors, while the results define the negative relationship between stock index XU100 and these factors.

Nevertheless, using advance statistics methods could provide significant contribution to the research (Amman et al., 1996). Safara, (2020) proposed a prediction model to forecast the consumers’ behaviour using machine learning methods. She used Five individual classifiers with Bagging and Boosting on online shopping dataset during the Covid-19 epidemic. The findings indicated that the decision tree model ensembles with Bagging achieved the best prediction of consumer behaviour with the accuracy of 95.3%. Alsayed and Manzi, (2019) used the developed Monotonic Dependence Coefficient to detect the economic growth. Al Sayed et al., (2018) applied various diagnostic outlier’s methods to detect the extreme values of economic growth. Zaidi et al., (2017) applied an inversed function regression to minimize the error term of the estimation model. Alsayed and Manzi, (2018) used several robust estimators to model the changing of GDP per capita for developed countries. Zaidi, (2015) used the fixed effect model to control the variation in energy consumption at the economic growth in selected developing countries.

## Materials and Methods

### Data

In order to assess the behaviour of the Turkish stock market during the period of Covid-19 pandemic and Russian invasion, the data are collected from the beginning of the epidemic in Turkey at March 11, 2020 until the recent date of this research to include a time period of Russian invasion May 31, 2022. The Covid-19 factors; for the new daily confirmed cases are obtained from Republic of Turkey Ministry of Health (MHT, 2022). On the other hand, the dataset of the main index which representing the stock market exchange in Turkey BIST-100 (XU100) is obtained from the Central Bank of the Republic of Turkey (CBRT). The other variables are gathered from various data sources; foreign exchange rates from Bloomberg Terminal (2022) which is used as control variables, while credit default swap is obtained from Investing Database (2022). The XU100 includes the daily stock prices and is considered as the dependent variable in this experimental research, also we considered the changing of the stock market values of removing two zeros from the index in July 2020. In the analysis, the XU100 index are used at the closing price of the trading session, other included variable representing the country level economic are credit default swap, foreigner exchange rate USD/TL, and TL reference interest rate. The data for all variables include only the weekday observations, but the weekend, and official holidays are excluded from the analysis.

**Fig. 1:**
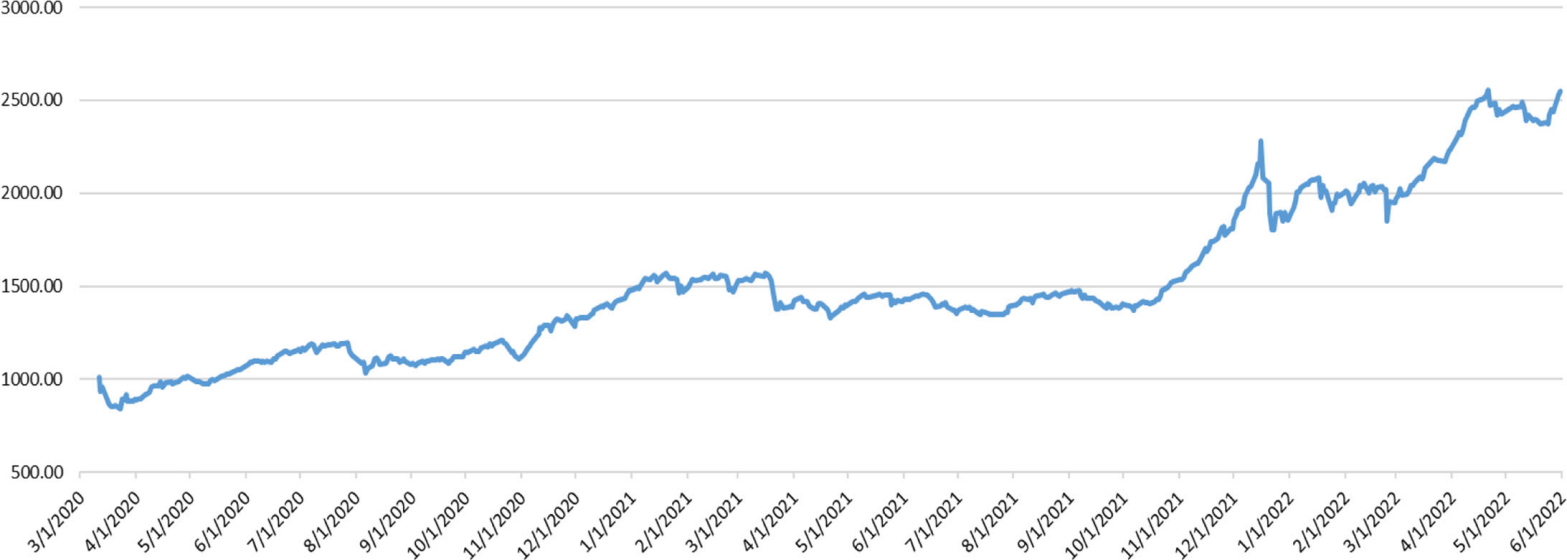
Turkish Stock Market Trend XU100

**Fig. 2:**
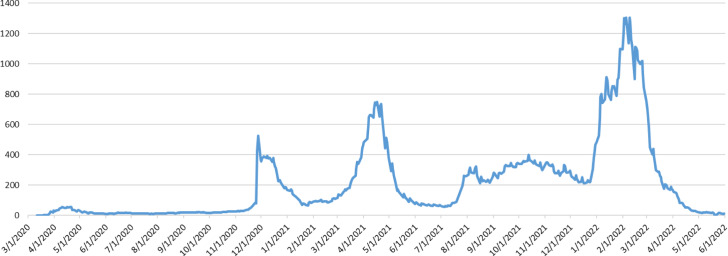
The Trend of new Covid-19 Cases per million in Turkey

Figure 1 illustrates that the XU100 index has been declined sharply at the beginning of the epidemic, then it increases slowly till it reaches to high level in early of 2022. In addition, Fig. 2 represents the daily new cases per million, it is clearly that the number of new cases in Turkey are not stable during the whole period as it is increased sharply at several points. Figures 1 and 2 suggest that stock market prices are negatively related to new daily confirmed cases caused by Covid-19. In contrast, the XU100 index price increased to the highest level during the period of this study to reach more than 2500 after the Russian invasion.

### Statistical Methods

In this section, we illustrate briefly the applied statistical methods in this research, which are used to investigate the effect of the Covid-19 epidemic on the stock market index XU100 in Turkey during the epidemic and the Russian invasion. we apply the current developed elastic-net (EN) regression based on empirical mode decomposition (EMD) which tackles the multicollinearity, nonstationary, and nonlinearity problems (Al-Jawarneh, al, et. 2020). EN regression is penalized least squares estimator model, it analyses high-dimensional datasets and select the most significant predictors those influencing the response variable. It effectively shrinks coefficients similar to ridge regression, and it set some coefficients to exactly zero as the works of lasso regression (Zou & Hastie, 2005).

1 $$\widehat{\beta }= { {argmin}_{\beta }|\left|{\varvec{y}}-{\varvec{X}}\beta \right||}^{2}+ {{\lambda }_{2}|\left|\beta \right||}^{2}+{\lambda }_{1}{|\left|\beta \right||}_{1}$$

In addition, the EMD method adaptively decomposes the nonstationary and nonlinear original data signal into a finite set of orthogonal components, i.e. linearly independent components based on the local characteristic timescale of the data, those components called intrinsic mode functions (IMFs) and residual/trend component without changing in the time series period, while in economic the trend component could represent the trend of the variable in long run (Huang et al., 1998).

2 $${x}_{t}=\sum_{i=1}^{n}{IMF}_{i, t}+ {\varepsilon }_{n,t}$$

where *n* represent the number of IMF, $i$ represents the number of component, ${\varepsilon }_{n,t}$ represents the residual component. The IMF_s_ and the residual component ${\varepsilon }_{n,t}$ are linearly related to the original data *x*_*t*_.

The combination of EMD&EN addresses the problem of nonstationary and nonlinear datasets and can tackle the multicollinearity problem. Firstly, it works to decompose the original signals of datasets into a set of orthogonal IMF components and a residual component, then those decomposition components are used as orthogonal new predictor variables to be modelled with the response variable by the EN regression, where the latter could enhance the prediction accuracy by selecting the subset of the decomposed components with the strongest effect into the response variable.

Moreover, the cross-validation (C.V) method is used in order to select the appropriate level of flexibility with the applied methods to obtain the best λ component which minimize the MSE. To sum up, several criteria or goodness of fit are used to evaluate the best performance among the applied methods; such as coefficient of determination (R^2^), Root Mean Square Error (RMSE), and Mean Absolute Error (MAE) and mean absolute scaled error (MASE). Further, the presence of multicollinearity tends to increase the variance of estimated coefficients and leads to inaccurate estimations. It is tested by using variance inflation factor (VIF).

Nonetheless, the hypothesis we assume in this research is that the spread of the Covid-19 virus effects significantly into the stock market index, also the Russian invasion has a significant influence of changing in stock market index. The stock market price is modelled as a function of several factors as illustrated in Eq. 3.

$${XU100 }_{t} = {\alpha }_{t}+{\beta }_{1t} N.C.m+ {\beta }_{2t} {CDS+ \beta }_{3t} USDTL {+ \beta }_{4t} TLREF+ {\varepsilon }_{t} (3)$$

where *XU100* represents the stock market index at day *t* in Turkey, *N.C.m* is the new confirmed cases per million caused by Covid-19, credit default swap (CDS), foreigner exchange rate USD/TL (USDTL), TL reference interest rate (TLREF) and *ε*_*t*_ is the error term.

**Fig. 3:**
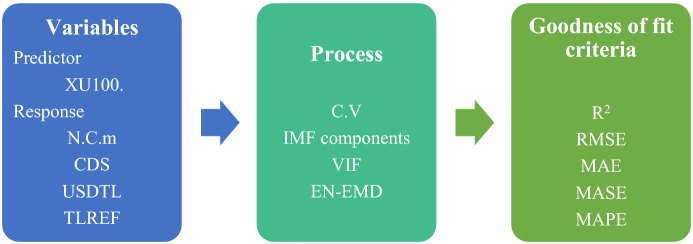
Flow Chart of the Analysis Process

Nevertheless, the analysis of this research could be summarized as shown in Fig. 3. All variables are the standardization. The stationarity of data is checked by using the Augmented Dickey-Fuller and KPSS test. However, regarding the EN-EMD analysis, we construct the IMF components by the EMD approach, then the multicollinearity is tested among the IMF components. Finally, the goodness of fit criteria is calculated for the estimated model.

## Results and Discussion

Firstly, we estimated the model using the elastic net regression for the test data as illustrated in Table 1. However, *new cases per million* have a positive significant impact on the XU100 index. That could be explained as the new Covid-19 cases were recorded with a low number until November 24, 2020, after that, there were a two announcement by the Turkish Health Ministry about Covid-19; first announcement is about the recording of the new daily Covid-19 cases to includes not only the hospitalized or inpatients but also the outpatients in the recording which increased sharply the recording of new Covid-19 cases in Turkey. Along with the second announcement which is about the number of daily patients which included only the recorded hospitalized patients. In additional to that, the effect of country level economic factors shows that foreigner exchange rate USD/TL has a positive significant impact on the XU100 index, but credit default swap and TL reference interest rate have a negative impact on the values of XU100 index. That could be explained the increase in foreigner exchange and monetary policy causes an increase in the XU100 index implying that Turkey declared various measures such as increasing interest rates to stabilize the economy. The results of this research are in line with the finding of Kartal et al., (2021) study, as they concluded significant positive effects of the Covid-19 towards the XU100 stock market index, and it is similar to the results study of (Özkan, 2020; Yağli 2020), as their finding shows that the volatility of stock market index increased rapidly during the Covid-19 epidemic. However, other studies shown that the Covid-19 epidemic has a negative impact on almost all economic sectors (Özkan, 2020).

**Table 1:** The estimated model by elastic regression

Elastic regression	Coefficients	Criteria	Value
β_1_	0.245	RMSE	4.285
β_2_	−0.681	MAE	3.729
β_3_	0.553	MAPE	3.903
β_4_	−0.212	R^2^	0.786

Where β_1_, β_2_, β_3_, & β_4_, indices to the *new cases per million, credit default swap, foreigner exchange rate USD/TL (USDTL), and TL reference interest rate (TLREF) respectively*

**Fig. 4:**
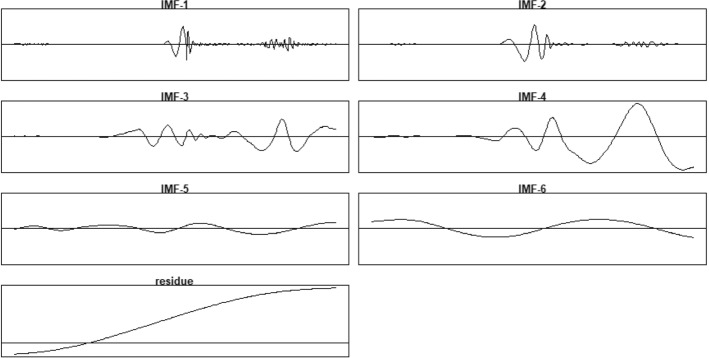
Decompose the Nonstationary of *new cases per million* Time-Series by EMD into IMF Components

On the other hand, it is noteworthy from Fig. 2, and from the statistical stationarity test that the variable of new cases per million is nonstationary and nonlinear, as it does not have a constant changing rate over time. This finding is comparable to that in the study of Kartal et al., (2021), who used two nonlinear techniques; NARDL and MSR to overcome the nonlinearity of the impacts of Covid-19 on XU100. To overcome those obstacles in this research, we model the XU100 with the variable of new cases per million by applying the developed EMD-EN approach to have a clearer idea of that relationship by dividing the variability of the new cases per million into decomposition components (IMFs). Figure 4 shows that the new cases per million have been decomposed into seven components of IMFs of which each component has distinct characteristics at different time scales. As the disparities are clear from IMF_1_ to IMF_6_ in terms of increasing the wavelength, but reducing the frequency and amplitude. However, the residue is slowly varying around the long-term average. Therefore, it is treated as the long-term trend during the changing of the number of new cases. While each sharp up or down of low frequency of IMF_5,_ IMF_6,_ IMF_7_ components are corresponding to significant events such as the waves of the Covid-19 epidemic, lockdown or a certain policy by the government, etc., and it could be the main reason for the dramatic variability of new cases rate in the medium and long term. That also could be related to the two announcement by the Turkish Health Ministry about Covid-19 regarding the recording of the new Covid-19 cases and the daily death cases in Turkey on November 24, 2020 (MHT, 2020). However, the changing up and down in the curves of the high-frequency components; IMF_1,_ IMF_2,_ IMF_3_, and IMF_4_ should be explained as normal fluctuations events of the Covid-19 epidemic in the variability of the new cases rate or due to some policies in the short term impact into XU100 index.

**Fig. 5:**
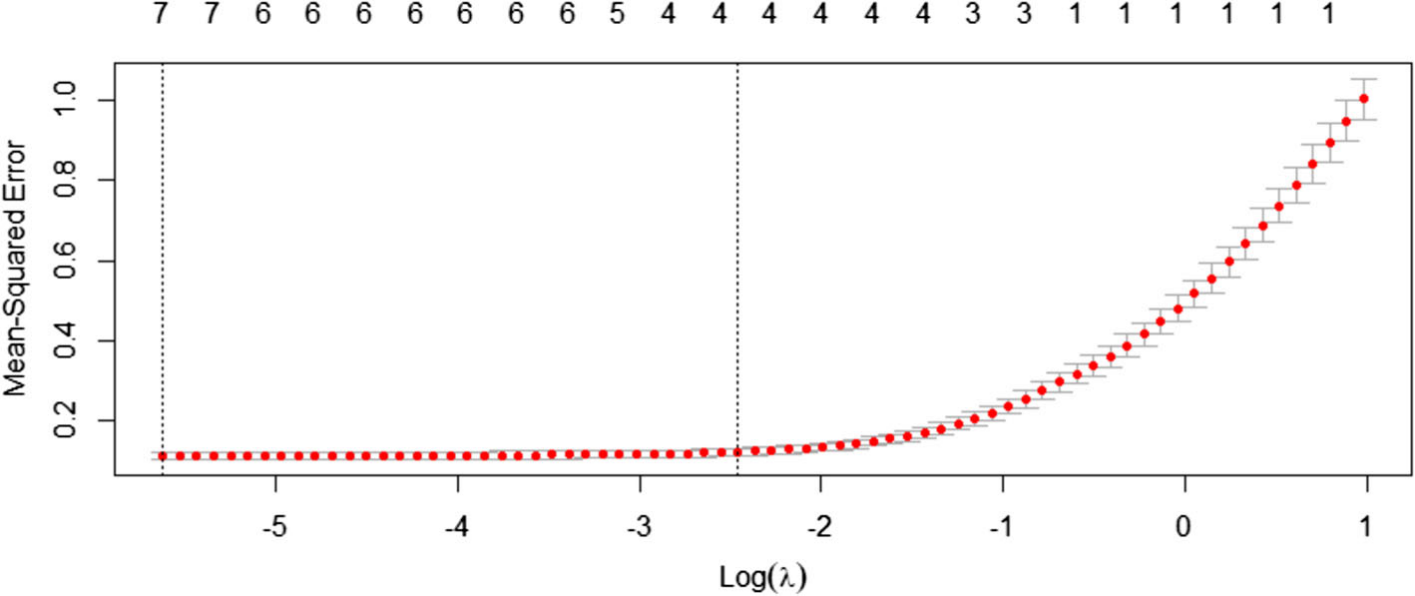
C.V Technique for EN-EMD Model

Further, Table 2 provides the results of VIF multicollinearity test among all the orthogonal IMF components, the finding illustrates that there is no multicollinearity among all constructed IMFs and residue components, as all values are less than 10. Besides, Fig. 5 represents the C.V for the MSE versus the Log (λ) to model the EMD-EN. The upper horizontal line of the plot represents the numbers of nonzero regression coefficients for a given Log (λ) which suggest having the four low-frequency of IMF components.

**Table 2:** Results of variance inflation factors multicollinearity test

IMF1	IMF2	IMF3	IMF4	IMF5	IMF6	Residue
1.94	1.43	1.56	1.77	1.23	1.42	1.71

**Fig. 6:**
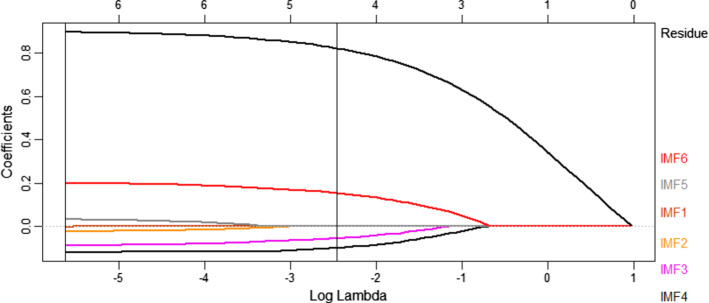
Coefficients Estimation by EMD-EN Model at Alpha 0.34

Apart from this, Fig. 6 illustrates the relationship between the log (λ) and the nonzero estimated coefficients by EMD-EN. The coefficients are estimated according to the value of the minimum λ which includes all IMF components at different significant levels of impact on the XU100 index. The trend or residue component has significant effects on XU100 and greater than the other components. Meanwhile, the IMF_1_ component is only significant at the 1st standard error of λ. Those findings of EMD approach are in line with the study of (Dai Z. & Zhu H. 2020), for the forecasting of the stock market return at the long-run trend by applying the sum-of-the-parts method and ensemble empirical mode decomposition.

Above all, the results provide very significant predictability for XU100 index both in economic and statistical terms, as all the IMFs coefficients are included in the final model. Table 3 illustrates the estimated coefficients of the IMFs and residual components of the new cases per million into the XU100 index by using the minimum λ, as it outperforms the first standard error of λ based on the lowest value of RMSE. Interestingly, some of the IMFs estimated coefficients significantly affect the XU100 negatively but others affect it positively. It can be observed that the high frequencies IMF’s coefficients (IMF_1_to IMF_4_) are affecting negatively the XU100 index gradually, while the lower frequencies IMFs coefficients (IMF_5_, IMF_6_ and trend component) have a significant positive effect on XU100 index and are relatively higher than that in the high frequency of IMFs. That is since IMFs of lower-frequencies are very smooth, and thus, it is very likely that they co-vary. In other words, long-term fluctuations; IMF_6_ and IMF_7_ of the *new cases per million* variable exhibited the strongest effects on the XU100 Index. The trend represents the major trend of a number of new cases in the long-term. It is noteworthy that the signs of the low frequency coefficients IMF_5_, IMF_6_, and the trend component are similar to the results in Table 1. So the IMF_5_, IMF_6_ components and residue have abundant economic meanings and reveal some new features of XU100 index. Furthermore, Table 3 provides the estimated coefficients for the IMF Components and Goodness of Fit Criteria.

**Table 3:** Estimated coefficients for the IMF components and goodness of fit criteria

Components	Coefficients	Criteria	Value
IMF_1_	−0.017	RMSE	0.439
IMF_2_	−0.035	MAE	0.350
IMF_3_	−0.071	MAPE	0.324
IMF_4_	−0.120	MASE	4.549
IMF_5_	0.072	R^2^	0.822
IMF_6_	0.181		
Residue	0.789		

### Robustness Check

We performed the robustness check by applying a nonlinear techniques Markov switching regression (MSR) following the study Kartal et al., (2021) to examine the impacts of covid-19 factors and economic factors at country level for stock market index XU100 in Turkey. The findings are consistent with the finding in the Table 2, which shows significant coefficients for all variables in this study, and a negative impact of credit default swap and TL reference interest rate into XU100 index.

## Conclusion

As a result of the coronavirus epidemic and Russian invasion, the financial markets suffered from uncertainty, stock markets are collapsed, and market volatility climbed rapidly. Although several attempts have been made to understand the empirical impact of Covid-19 on the economic situation, but it is still inexplicitly in most cases. This study aims to investigate the impacts of the coronavirus pandemic and Russian invasion on the stock market index in Turkey by applying the developed approach of the elastic-net regression based on empirical mode decomposition to tackle the nonstationary and nonlinearity. The data are collected from the beginning of the epidemic in Turkey from March 11, 2020 until May 31, 2022. The findings of this study provide significant predictability for XU100 index by positive significant impact of new Covid-19 cases per million. Moreover, the effect of country level economic factors shows that foreigner exchange rate USD/TL has a positive significant impact on the XU100 index, but credit default swap and TL reference interest rate have a negative impact on the values of XU100 index. In addition, the findings of second part of analysis which related to EMD-EN approach reveal that the variable of new cases per million can be decomposed into seven components with distinct characteristics to overcome the nonstationary and nonlinearity. The higher and lower frequencies IMFs coefficients are affecting negatively and positively the XU100 index, respectively. That could be related to the two announcement by the Turkish Health Ministry about Covid-19 regarding the recording of the new Covid-19 cases and the daily death cases in Turkey on November 24, 2020 (MHT, 2020). Thus, the elastic-net regression based on empirical mode decomposition has relative great regularization and selected predictor variables, also it decomposes the nonstationary and nonlinearity data to reveal an accurate trend of the effects on XU100 index.

## Data Availability

The used data of this study are available in Bloomberg website https://evds2.tcmb.gov.tr/index.php?/evds/serieMarket and the Central Bank of Republic of Turkey at and Turkish Ministry of Health at https://covid19.saglik.gov.tr.
